# Treatment of Near-Infrared Photodynamic Therapy Using a Liposomally Formulated Indocyanine Green Derivative for Squamous Cell Carcinoma

**DOI:** 10.1371/journal.pone.0122849

**Published:** 2015-04-07

**Authors:** Tetsuro Maruyama, Yasunori Akutsu, Akiko Suganami, Yutaka Tamura, Hiromichi Fujito, Tomoki Ouchi, Naoki Akanuma, Yuka Isozaki, Nobuyoshi Takeshita, Isamu Hoshino, Masaya Uesato, Taro Toyota, Hideki Hayashi, Hisahiro Matsubara

**Affiliations:** 1 Department of Frontier Surgery, Graduate School of Medicine, Chiba University, Chiba, Japan; 2 Department of Bioinformatics, Graduate School of Medicine, Chiba University, Chiba, Japan; 3 Department of Medical System Engineering, Faculty of Engineering, Chiba University, Chiba, Japan; 4 Division of Nanoscience, Graduate School of Advanced Integration Science, Chiba University, Chiba, Japan; 5 Department of Basic Science, Graduate School of Arts and Sciences, The University of Tokyo, Tokyo, Japan; 6 Center for Frontier Medical Engineering, Chiba University, Chiba, Japan; Winship Cancer Institute of Emory University, UNITED STATES

## Abstract

**Introduction:**

Photodynamic therapy (PDT) is a less invasive option for cancer treatment that has evolved through recent developments in nanotechnology. We have designed and synthesized a novel liposome system that includes an indocyanine green (ICG) derivative, ICG-C18, in its bilayer. In addition to its use as an optical imager to visualize blood, lymphatic, and bile flow, ICG has also been used as an optical sensitizer. In the present report, we evaluate the use of our novel liposome system, LP-ICG-C18, in PDT for squamous cell carcinoma in an autologous murine model.

**Materials and Methods:**

An excitation pulse beam (300 μJ/pulse) of a single band (800 nm) was used for sensitization. The cytotoxicity of the photodynamic therapy was evaluated in terms of cellular morphology changes, methyl thiazolyl tetrazolium (MTT) assay results, and terminal deoxynucleotidyl transferase-mediated deoxyuridine triphosphate-biotin nick end labeling (TUNEL) staining. We tested the enhanced permeability and retention effect of LP-ICG-C18 in tumor-bearing C3H/He mice using a near-infrared fluorescence imaging system and fluorescence microscopy. We also examined the antitumor effect of PDT by measuring tumor volume in tumor-bearing mice.

**Results:**

Cell death and apoptosis were only observed in the PDT group receiving LP-ICG-C18. LP-ICG-C18 itself had no cytotoxic activity and showed good biocompatibility. LP-ICG-C18 accumulated on the tumor 24 hours after injection and was retained for approximately 3 weeks. Tumor cell apoptosis following PDT with LP-ICG-C18 was also observed under optical microscopy, MTT assay, and TUNEL staining.

**Conclusion:**

These findings suggest that LP-ICG-C18 may be an effective intervening material in PDT for malignant disease.

## Introduction

It is widely known that nanoparticles are useful vehicles for targeting tumors and can serve as appropriate drug delivery tools because of the enhanced permeability retention (EPR) effect [1]. Various nanoparticles have been encapsulated or conjugated to photosensitizers for the purpose of cancer therapy, and have been used for daily treatments [2–9]. These benefits have primarily been achieved through recent developments in nanotechnology.

Indocyanine green (ICG) has a spectral absorption at approximately 780 nm and a high-intensity fluorescence emission at approximately 820 nm[10]. ICG has low toxicity and induces heat and singlet oxygen formation in response to near-infrared (NIR) light with a wavelength of 800 nm [11–14]. Because of these benefits, ICG has been used widely, both as an optical imager for the evaluation of liver function and sentinel node biopsies and as an optical sensitizer in photodynamic therapy (PDT) [10].

Several previous studies have discussed the use of liposomally formulated ICG (LP-ICG) in optical imaging and cancer treatment [15, 16]. However, conventional ICG can leak from the liposomal membrane of LP-ICG. Accordingly, we have designed and synthesized a novel NIR photoactivating probe, called ICG-C18, that is more hydrophobic than conventional ICG. In a previous study, LP-ICG-C18 yielded brilliant fluorescence images under the NIR-fluorescence imaging system in both *in vitro* and *in vivo* conditions [17].

Although the triad of surgery, chemotherapy, and radiotherapy is currently the standard treatment for esophageal cancer, the survival rate of patients with this disease is poor. PDT has recently been used in the treatment of esophageal cancer, oral cancer, skin cancer, and other types of cancer [18–22]. Moreover, many photosensitizers (PSs) have been reported to be used in PDT, such as porphyrin, chlorine, purpurin, phthalocyanine, and benzoporphyrin [23]. However, most PSs are not tumor selective, affecting normal tissue around the tumor in addition to the tumor itself. Therefore, it is difficult to irradiate the tumor directly in the esophagus.

In such cases, we believe that LP-ICG-C18 may be effective for both tumor imaging and tumor-selective PDT, as a consequence of the EPR effect. In the present study, we aimed to evaluate and assess the utility of LP-ICG-C18 in PDT for squamous cell carcinoma in mice, under both *in vitro* and *in vivo* conditions.

## Materials and Methods

### Cell culture

Murine squamous cell carcinoma SCCVII tumor cells were kindly provided as a gift from Professor Yuta Shibamoto (Department of Quantum Radiology, Nagoya City University, Nagoya, Japan) in 2007 and were used in this study. The characteristics of these tumor cells have been described fully in previous research [24]. SCCVII cells were maintained in Dulbecco’s modified Eagle’s medium (DMEM), supplemented with 10% fetal bovine serum and 100 units/mL of penicillin-streptomycin-neomycin solution. The cells were maintained at 37°C in a humidified incubator with 5% CO_2_.

### LP-ICG-C18 preparation and characterization

LP-ICG-C18 was prepared as previously described [17]. The average size of LP-ICG-C18 was 235 ± 101 nm, which is almost the same as that of LP-ICG (209 ± 79 nm). As previously reported [17], the peak wavelengths of absorbance of ICG, LP-ICG, and LP-ICG-C18 ranged from 780 to 800 nm, and the peak wavelengths of fluorescence ranged from 800 to 820 nm. Moreover, the absorbance and fluorescence intensities of LP-ICG-C18 were higher than those of ICG aqueous solution and LP-ICG. For use as a control, we additionally prepared liposomes (LP) without ICG or ICG-C18.

### Light source

In the present study, an excitation pulse beam (300 μJ/pulse) of a single band (800 nm) was used as a light source. The cell cultures were placed at a 2-cm distance from the light source and irradiated for 15 min (0.5 W/cm^2^). The mouse tumors were placed at a 2-cm distance from the light source and irradiated for 20 min per day (0.5 W/cm^2^).

### In vitro analysis: cellular uptake of LP-ICG-C18

We evaluated cellular uptake of LP-ICG-C18 and ICG. SCCVII cells (5 × 10^3^ cells) were seeded in 96-well cell culture plates with DMEM and maintained in the incubator at 37°C and 5% CO_2_ for 4 hours to allow attachment and growth. After the 4-hour incubation period, DMEM was removed from the culture plates. The following solutions were then added to different cell culture plates: ICG solution at concentrations of 1 μM, 10 μM, and 100 μM; LP-ICG-C18 solution at concentrations of 1 μM, 10 μM, and 100 μM; and saline. All of the cell culture plates were then incubated for an additional 24 hours at 37°C and 5% CO_2_. After this additional incubation, the solution was removed from each plate and all the plates were washed with phosphate-buffered saline (PBS). Thereafter, we examined the cells using a microscope (BX51, Olympus Corp., Tokyo, Japan) with a near-infrared filter set (ICG-A, Semrock, Inc, NY, USA) and an electron multiplying charge coupled device (EMCCD) monochrome camera (iXon-DU897E, Andor Technology Japan, Tokyo, Japan).

### In vitro analysis: PDT and cell viability assay

After the incubation period that was designed to allow attachment and growth (as described above), all of the cell culture plates were incubated for an additional 20 hours with only one of these solutions was added per plate: DMEM, LP solution, ICG solution (concentrations of 100 μM), or LP-ICG-C18 solution (concentrations of 100 μM). All of the culture plates were then washed twice with PBS, and DMEM was added. The cell cultures were placed at a 2-cm distance from the light source, irradiated for 15 min (0.5 W/cm^2^), and then incubated in the absence of light. Ninety-six hours after irradiation, cell morphology was observed using a phase-contrast microscope (Axiovert 200, Carl Zeiss Co. Ltd, Oberkochen, Germany). In addition, cell viability was evaluated at 0, 24, 48, 72, and 96 hours after irradiation, using cell counting kit-8 (CCK-8, Dojindo Laboratories, Kumamoto, Japan). Absorbance was measured at 450 nm with a microplate reader (xMark Microplate Absorbance Spectrophotometer, BIO-RAD Co. Ltd, California, United States). We also evaluated cell apoptosis through TUNEL staining (MK500, in situ Apoptosis Detection Kit, Takara Bio Inc., Shiga, Japan) 96 hours after irradiation. TUNEL-positive cells were quantified through manual counting.

### In vivo analysis: Tumor-bearing mice

SCCVII cells (1 × 10^5^ cells per mouse) were inoculated subcutaneously into the posterior portion of the right thigh of female CH3/He mice (average body weight, 19 g; Japan SLC Inc., Shizuoka, Japan). The mice were selected to undergo the experiment after the tumors grew to approximately 3 mm in diameter. The body and leg hair of each mouse were shaved to enable observation.

Subsequently, mice were injected with either saline, LP, or LP-ICG-C18, forming 3 experimental groups that are described later in this article. All solutions (saline, LP, and LP-ICG-C18) were injected through the tail vein.

Isoflurane was used to anesthetize the mice. An anesthetic vaporizer was used to deliver the isoflurane as a known percentage (3% for maintenance, 5% for introduction) in oxygen. All mice were sacrificed using cervical dislocation, as performed by a skillful researcher.

All of our animal studies were approved by the Animal Care and Use Committee at Chiba University (Permit Number: animal 25–70), and were performed in compliance with guidelines of our facility.

### In vivo analysis: EPR effect and LP-ICG-C18

For this experiment, 6 tumor-bearing mice were divided into two groups. In the first group, 3 mice were injected with 100 μl saline. In the second group, the remaining 3 mice were injected with 100 μl LP-ICG-C18 solution at concentrations of 100 μM. The mice in each group were observed every 24 hours, and photons were counted for 4 weeks using an NIR-fluorescence imaging system (IVIS lumina II, Caliper, MA, USA) with an XFL-HR Fluorescence Filter Option High Range (720–840 nm) detection filter.

Two further groups of additional tumor-bearing mice were prepared, each of which included 3 mice. The first group was injected with 100 μl saline and the second group was injected with 100 μl LP-ICG-C18 solution at a concentration of 100 μM. The tumors and other organs (brain, heart, lung, liver, kidney, spleen, stomach, small intestine, and colon) of these mice were removed 24 hours after injection. The tumors were cut in half—one half was frozen and the other was fixed in formalin and stained with hematoxylin and eosin (HE). A cryostat was used to cut the frozen tumor into 5-μm-thick slices, which were examined with a fluorescence microscope. The organs were examined using an NIR-fluorescence imaging system.

### In vivo analysis: toxicity to major vital organs

Four days after the injection of LP-ICG-C18 and irradiation, four mice were sacrificed and their organs (lung, liver, kidney, spleen, and small intestine) were stained with HE to check for toxicity.

### In vivo analysis: evaluation of temperature in the tumor following PDT

For this experiment, tumor-bearing mice were randomly divided into two groups (3 mice per group). The mice in group A were injected with 100 μl saline and those in group B were injected with 100 μl LP-ICG-C18 solution at a concentration of 100 μM. Five days after these injections, the mice’s tumors were irradiated. A thermometer with a needle probe (AMS-800, Adachi Keiki Corp., Tokyo, Japan) was used to assess the temperature at the center of the tumor, as well as the temperature inside of the rectum.

### In vivo analysis: antitumor effect of PDT with LP-ICG-C18

For this experiment, tumor-bearing mice were randomly divided into six groups (8 mice per group). In group 1, the mice were injected with saline and were not irradiated. In group 2, the mice were injected with saline and were irradiated for 5 days. In group 3, the mice were injected with saline and were irradiated for 4 weeks (5 days per week). In group 4, the mice were injected with LP-ICG-C18 and were not irradiated. In group 5, the mice were injected with LP-ICG-C18 and were irradiated for 5 days. In group 6, the mice were injected with LP-ICG-C18 and were irradiated for 4 weeks (5 days per week). A 100-μl LP-ICG-C18 solution (concentration of 100 μM) or saline was injected into each mouse. Seventy-two hours after injection, each of the mice in groups 2, 3, 5, and 6 underwent tumor irradiation for 20 min per day (0.5 W/cm^2^). During these sessions, the tumor was placed at a 2-cm distance from the light source. The tumor size was measured on alternate days for 4 weeks using calipers. The tumor volume was calculated as *D × d* ^*2*^ */2*, where *D* was the longest diameter and *d* was the shortest diameter.

In the subsequent histological analysis, the tumors of the mice in groups 1–6 were removed on day 28 and stained with HE. The slices that had been obtained were then examined using optical microscopy. Further, we used TUNEL staining to evaluate cell apoptosis in the treated tumors. TUNEL-positive cells were quantified through manual counting.

### Statistical analysis

Quantitative data are expressed as means ± standard deviations. Statistical comparisons were performed using analysis of variance and Student’s *t*-test and Mann-Whitney U test. P-values <0.05 were considered statistically significant.

## Results

### In vitro analysis: cellular uptake of LP-ICG-C18

**Fig 1:**
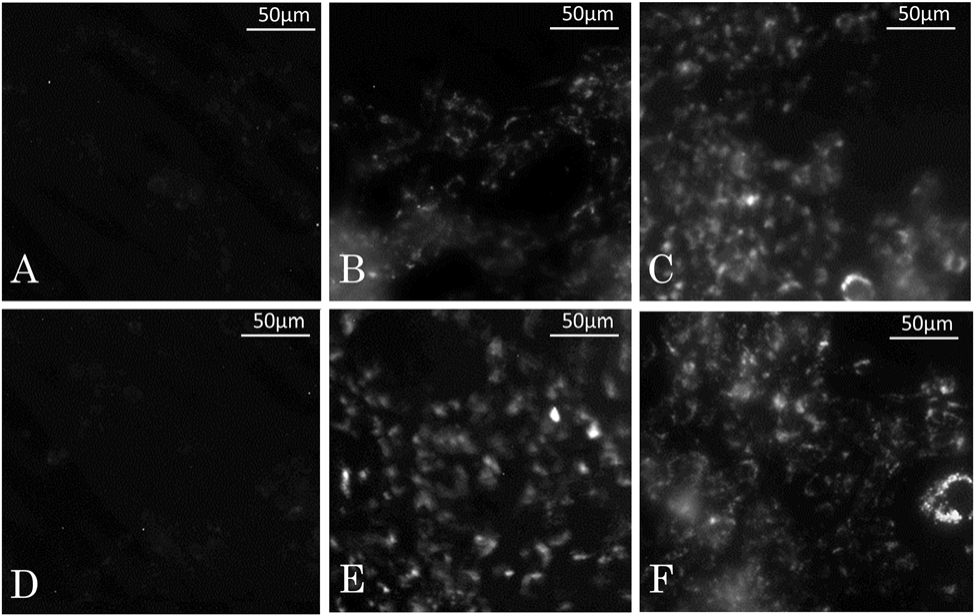
Fluorescence microscopy image of SCCVII cells incubated 24 hours after adding LP-ICG-C18 or ICG. (A) ICG 1 μM. (B) ICG 10 μM. (C) ICG 100 μM. (D) LP-ICG-C18 1 μM. (E) LP-ICG-C18 10 μM. (F) LP-ICG-C18 100 μM.

Twenty-four hours after they had been added to the cell culture plates, we evaluated the cellular uptake of ICG (Fig 1A, 1B and 1C) and LP-ICG-C18 (Fig 1D, 1E and 1F) in SCCVII cells. The control plates did not show any fluorescence when viewed under a fluorescence microscope. In the plates with ICG and LP-ICG-C18 solutions at concentrations of 1 μM, the cells had very weak fluorescence (Fig 1A and 1D). In the plates with ICG and LP-ICG-C18 solutions at concentrations of 10 and 100 μM, the cells exhibited a similar extent of fluorescence (Fig 1B, 1C, 1E and 1F).

### In vitro analysis: cytotoxicity of PDT with LP-ICG-C18

**Fig 2:**
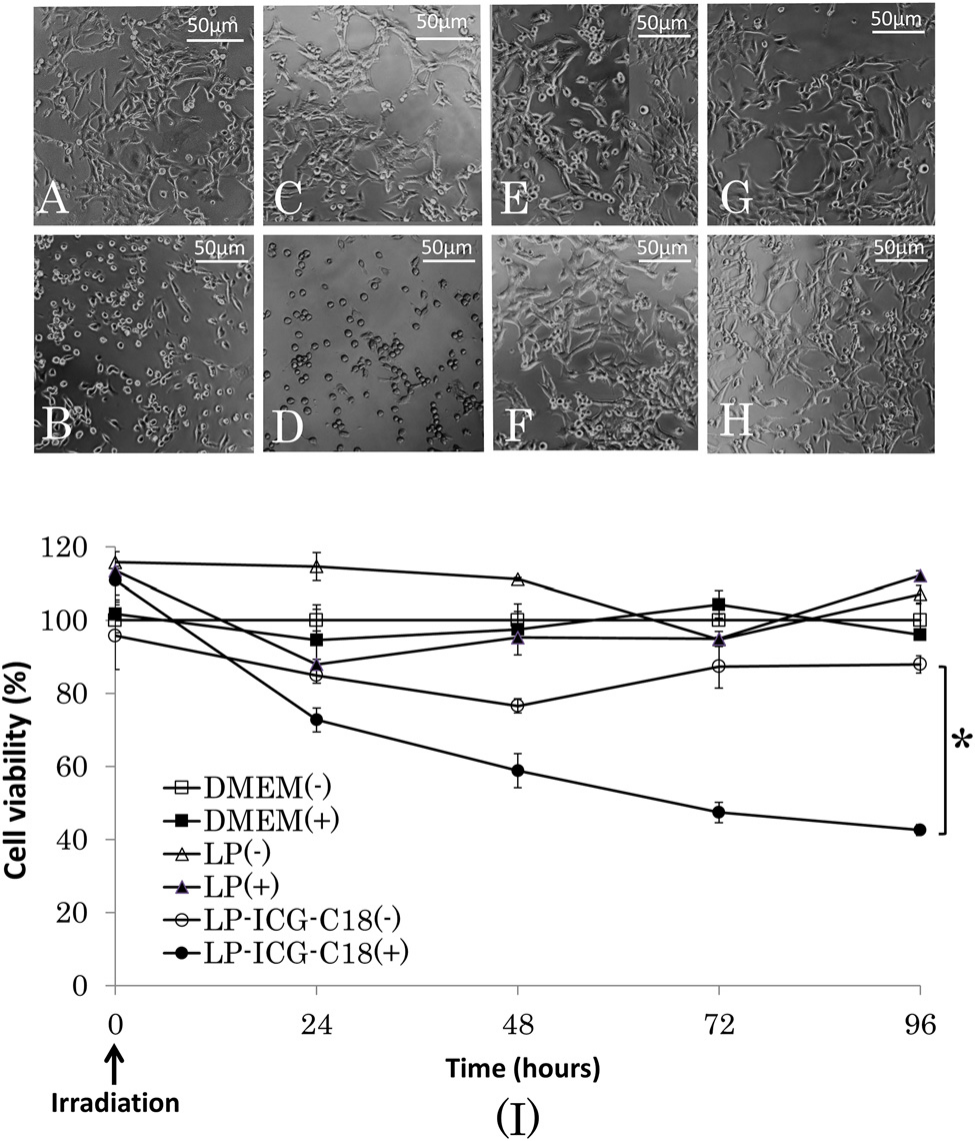
The cell morphology 96 hours after irradiation. (A) LP-ICG-C18 without irradiation. (B) LP-ICG-C18 with irradiation. (C) ICG without irradiation. (D) ICG with irradiation. (E) DMEM without irradiation. (F) DMEM with irradiation. (G) LP without irradiation. (H) LP with irradiation. (I) In vitro cytotoxicity comparison with DMEM without irradiation. “(-)” is without irradiation and “(+)” is with irradiation. “*” represents P<0.05 since 96 hours after irradiation. Data are presented as mean ± SD (n = 3).

INinety-six hours after irradiation, we assessed cell morphology in each cell culture (Fig 2; A: LP-ICG-C18 without irradiation, B: LP-ICG-C18 with irradiation, C: ICG without irradiation, D: ICG with irradiation, E: DMEM without irradiation, F: LP without irradiation, G: DMEM with irradiation, H: LP with irradiation). There were obvious changes in cell morphology in the cultures that had been subjected to LP-ICG-C18 with irradiation (Fig 2B). The cells were shrunken, round, and floating in the solution. However, there were no changes in cell morphology in the cultures that had been subjected to LP-ICG-C18 without irradiation (Fig 2A), suggesting that addition of LP-ICG-C18 alone did not cause any cytotoxicity and showed good biocompatibility. On the other hand, there were obvious changes in cell morphology in the cultures that had been subjected to ICG with irradiation (Fig 2D) and no changes in cell morphology in the cultures that had been subjected to ICG without irradiation (Fig 2C).

No changes in cell morphology were noted in the cultures subjected to DMEM without irradiation, DMEM with irradiation, LP without irradiation, or LP with irradiation (Fig 2E, 2F, 2G and 2H). The cells were spindle-shaped and adhered to the surface of the culture plate. These results indicated that significant cytotoxicity did not result from the addition of LP, irradiation, or both.

Fig 2I shows cell viability results, as measured using the MTT assay ([–]: without irradiation, [+]: with irradiation). A significant difference was observed when comparing the cells that were subjected to LP-ICG-C18 with and without irradiation [LP-ICG-C18 (+) and LP-ICG-C18 (-); P<0.01]. However, no significant cytotoxicity was observed in any of the following groups: DMEM with irradiation, DMEM without irradiation, LP with irradiation, LP without irradiation, and LP-ICG-C18 without irradiation.

**Fig 3:**
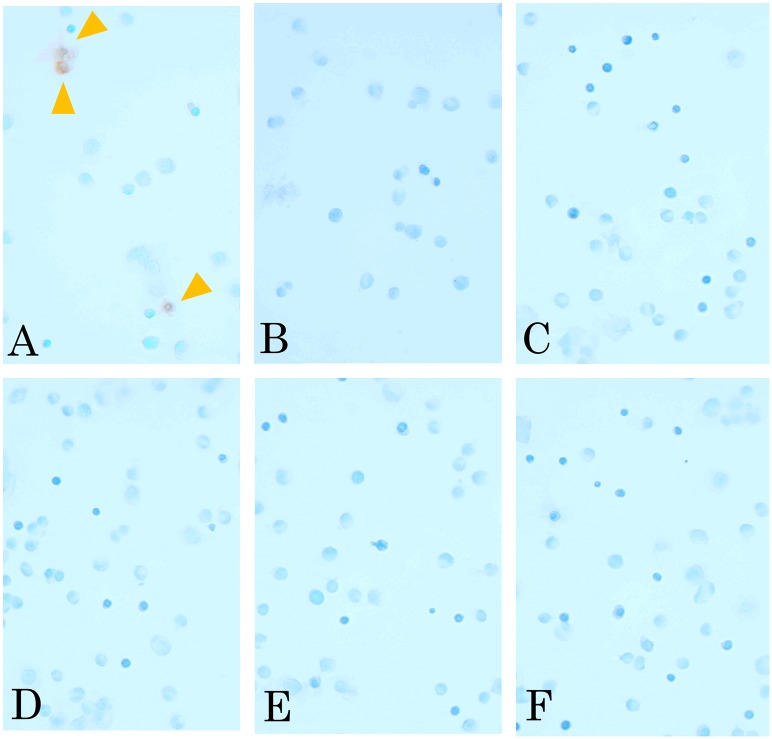
TUNEL staining of incubated SCCVII cells. (A) LP-ICG-C18 with irradiation. (B) DMEM without irradiation. (C) DMEM with irradiation. (D) LP without irradiation. (E) LP with irradiation. (F) LP-ICG-C18 without irradiation.

In contrast, TUNEL staining was also performed for each experimental group. Apoptotic cells were detected only in the LP-ICG-C18 group with irradiation (Fig 3A). Quantification indicated a 6.15% concentration of TUNEL-positive cells. TUNEL-positive cells were not observed in any of the other groups (Fig 3, B: DMEM without irradiation, C: DMEM with irradiation, D: LP without irradiation, E: LP with irradiation, and F: LP-ICG-C18 without irradiation).

### In vivo analysis: accumulation of LP-ICG in tumor-bearing mice

**Fig 4:**
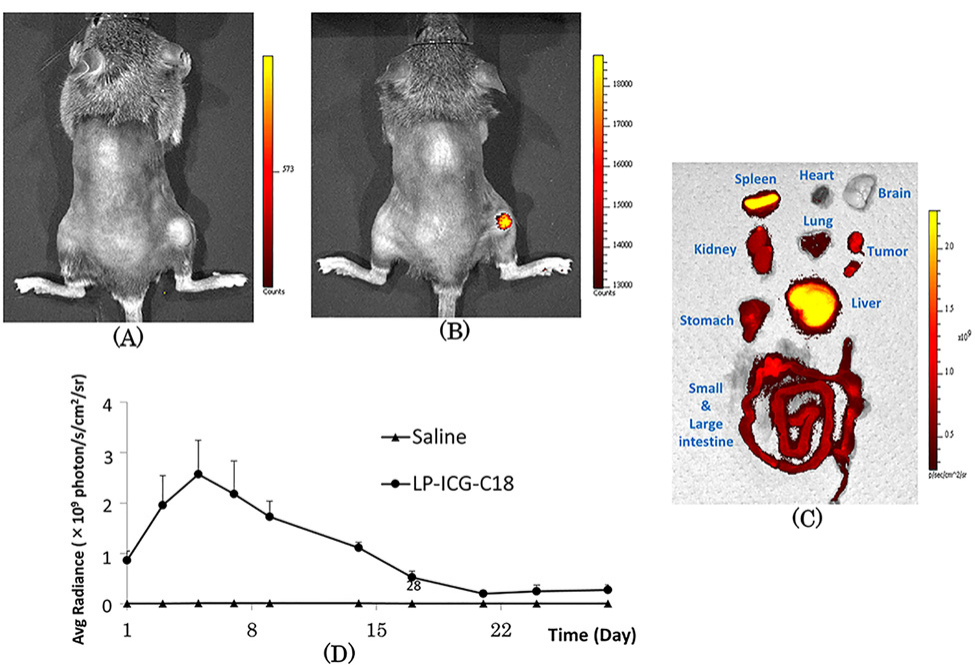
NIR fluorescence images of tumor bearing mice 24 hours after injection of (A) saline and (B) LP-ICG-C18. (C) NIR fluorescence images of the organs 24 hours after injection of LP-ICG-C18. (D) Photon count of tumor bearing mice.

Fig 4 shows the accumulation of LP-ICG-C18 in the murine body 24 hours after injection. Fig 4A presents a case in which saline was injected, while Fig 4B presents a case in which LP-ICG-C18 was injected. Remarkable accumulation of LP-ICG-C18 was observed in the tumor. On the other hand, accumulation of LP-ICG-C18 was also observed in the liver and spleen (Fig 4C). As assessed using the IVIS lumina II system (Fig 4D), the photon count in the tumor peaked 5 days after injection in the LP-ICG-C18 group. Thereafter, the photon count gradually decreased to a plateau during the 21 days after injection.

**Fig 5:**
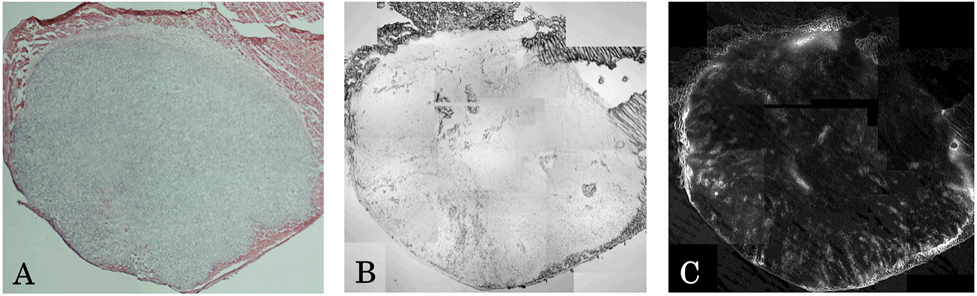
Image of resected tumor of mice in (A) HE staining, (B) Normal light image of fluorescence microscope, and (C) Fluorescence image of fluorescence microscope.

The removed tumor was also examined by optical and fluorescence microscopy. Fig 5A shows the tumor as stained with HE and observed during optical microscopy. Fig 5B and 5C show the tumor as observed during fluorescence microscopy (Fig 5B and 5C). As the results, the intensity of fluorescence was mostly strong at the surface of the tumor, but was weak within the tumor.

### In vivo analysis: toxicity to major vital organs

We performed microscopic observations in 4 days after LP-ICG-C18 injection. However, irradiation did not reveal any abnormalities in the major vital organs, such as lung, liver, kidney, spleen, and small intestine.

### In vivo analysis: evaluation of temperature in the tumor following PDT

**Fig 6:**
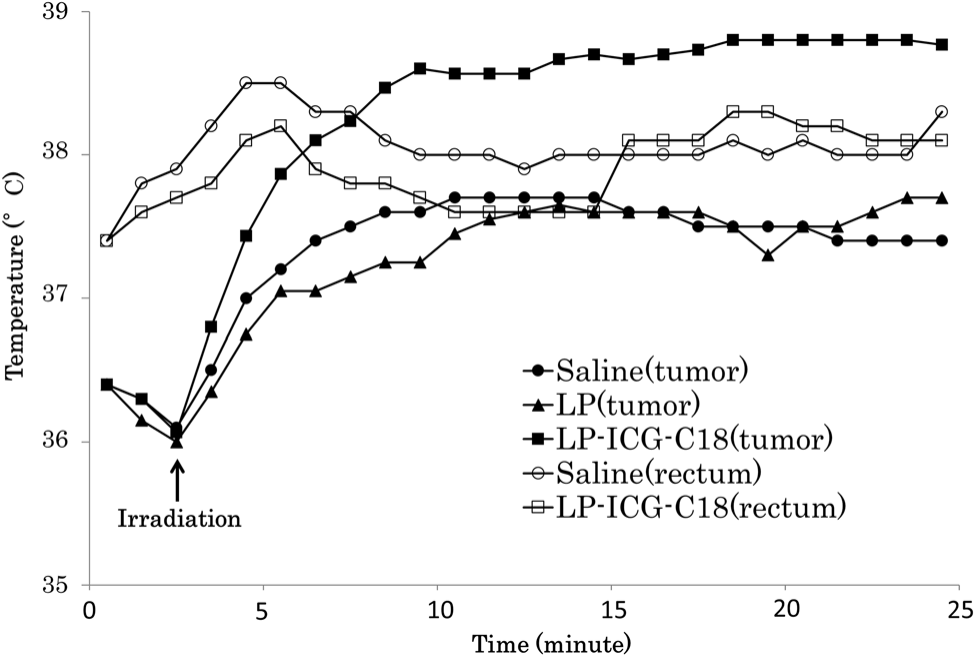
The transition of temperature in the tumor and rectum during irradiation.

Fig 6 shows the transitions of temperature at the center of the tumor and the rectum in response to PDT. The temperatures in the tumors of mice injected with LP-ICG-C18 increased by 3°C after irradiation, and reached a plateau after 10 min. However, the tumor temperature in the saline and LP groups only increased by 1.5°C after irradiation. Rectal temperature remained stable during irradiation. Therefore, PDT with LP-ICG-C18 only resulted in temperature increases in the tumor and may thus serve as a suitable tumor-specific treatment.

### In vivo analysis: antitumor effect of PDT with LP-ICG-C18

**Fig 7:**
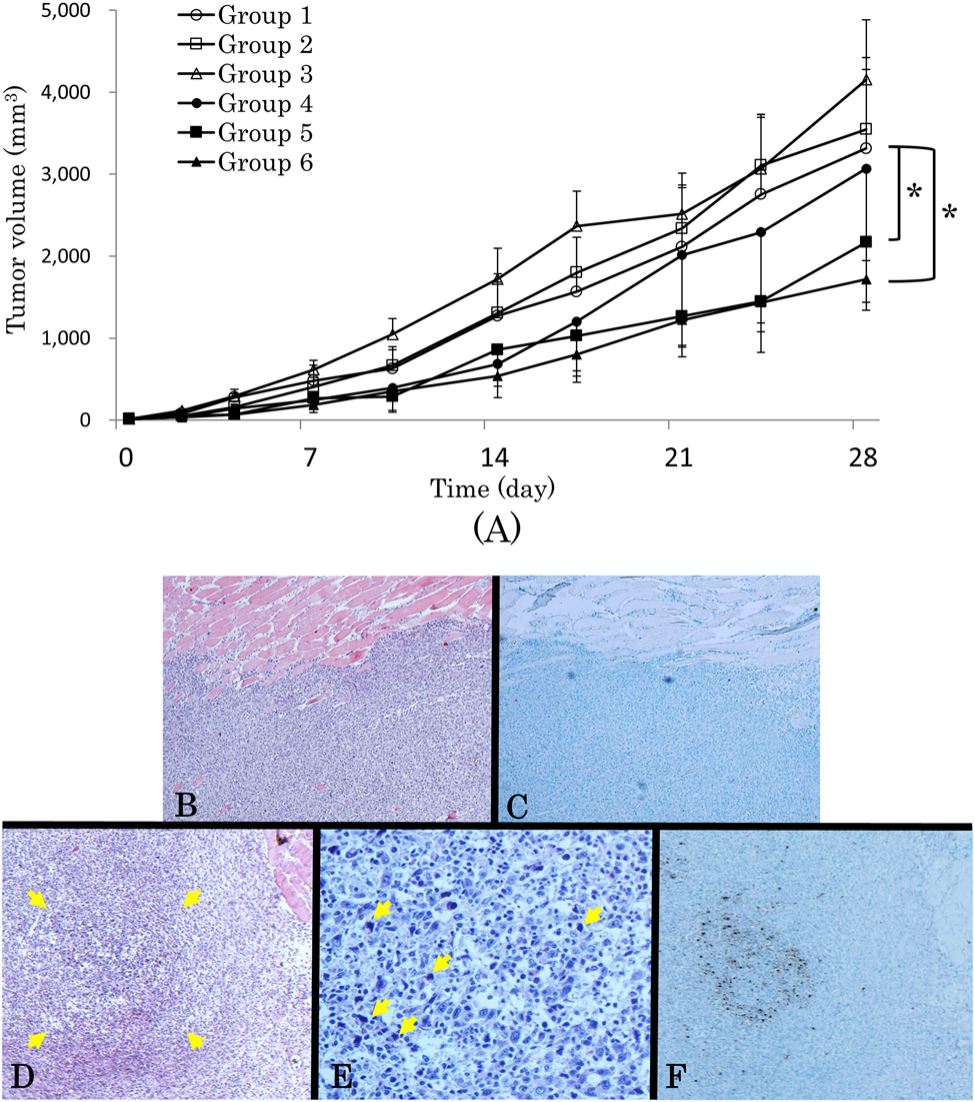
Antitumor effect of LP-ICG-C18 in SCCVII subcutaneous mice model. (A) Group 1: Injection of saline without irradiation, Group 2: Injection of saline with irradiation for 5 days. Group 3: Injection of saline with irradiation for 4 weeks (5 days in a week). Group 4: Injection of LP-ICG-C18 without irradiation. Group 5: Injection of LP-ICG-C18 with irradiation for 5 days. Group 6: Injection of LP-ICG-C18 with irradiation for 4 weeks (5 days in a week). “*” represents P<0.05 at 28 days. (B) HE staining of the tumor of Group 2. (C) TUNEL staining in the same place as (B). (D) HE staining of the tumor of Group 5. (E) High magnification images in necrotic area. (F) TUNEL staining in the same place as (D).

As shown in Fig 7A, tumor inhibition was not observed in groups 1 (saline without irradiation), 2 (saline with 5-day irradiation), 3 (saline with 4-week irradiation), and 4 (LP-ICG-C18 without irradiation). In those groups, the tumor volume increased rapidly; the mean volume was greater than 3000 mm^3^ on day 28. However, in groups 5 (LP-ICG-C18 with 5-day irradiation) and 6 (LP-ICG-C18 with 4-week irradiation), tumor growth was significantly suppressed. At the end of the experiment (day 28), the mean volumes were 2169.6 ± 689.6 mm^3^ in group 5 and 1717.3 ± 378.0 mm^3^ in group 6. Significant differences in tumor volume were observed between groups 1 and 5 and between groups 1 and 6 (both, P<0.01 by Student’s t-test). Although they were not significant by Mann-Whitney U test, there was a possibility of lack of statistical power because of small number of the mice. This result demonstrated that PDT by using LP-ICG-C18 is effective for tumor treatment *in vivo*, as further supported by our *in vitro* experiment described above, whereas LP-ICG-C18 alone and irradiation alone did not have any antitumor effect. Moreover, we performed HE staining and TUNEL staining of the excised tumor specimens from the saline without irradiation group and the LP-ICG-C18 with irradiation group. In the mice injected with saline without irradiation, a large number of tumor cells were viable and no apoptotic cells were detected (Fig 7B and 7C). However, in the tumors of the mice that received LP-ICG-C18 with irradiation, a large necrotic area was observed in Fig 7D (surrounded with an arrow) and apoptotic cells were detected in that area (Fig 7E). Quantification indicated a 1.62% concentration of TUNEL-positive cells (Fig 7F).

## Discussion

In the present study, we observed that LP-ICG-C18 was incorporated into cells to the same extent as ICG. However, the mechanism behind the cellular uptake of LP-ICG-C18 is still unknown and it remains necessary to uncover its precise details. Mechanisms of cellular uptake have been evaluated for several nanoparticles[11, 25], and have been reported to involve endocytosis, as demonstrated through the use of specific inhibitors for endocytosis [26–28]. Furthermore, researchers have recently reported a novel and efficient method that can be used to evaluate the cellular uptake of nanoparticles [29]. Therefore, future studies will be able to determine the actual mechanisms involved in the cellular uptake of LP-ICG-C18 by using such techniques.

ICG was recently reported to induce photo-oxidative cell killing through the generation of singlet oxygen and heat, whereas PDT with ICG was found to induce apoptosis and cell cycle arrest [13, 30–32]. LP-ICG-C18 resembles a previously investigated substance that is known as LP-iDOPE (liposomally formulated 1, 2-dioleoyl-sn-glycero-3-phosphoethanolamine conjugated ICG). Suganami et al. revealed that the physical properties of LP-iDOPE and ICG are similar, in the sense that irradiation of either substance produces singlet oxygen and heat [10]. Therefore, we suggest that PDT with LP-ICG-C18 induces cell apoptosis by generating singlet oxygen and heat. The results of our cell morphology examinations, cell viability assay (Fig 2), and TUNEL staining (Fig 3) support this suggestion. Furthermore, we noted that the cellular uptake of LP-ICG-C18 occurred easily at a concentration of 10 μM, and that PDT with LP-ICG-C18 induced cell apoptosis.

LP-ICG-C18 accumulated in the tumors of tumor-bearing mice as a consequence of the EPR effect. Although many reports have evaluated the EPR effect in tumor-bearing mice, few have investigated the length of the time period during which nanoparticles accumulate in the tumor. In the present study, the concentration of LP-ICG-C18 in the tumor peaked at 5 days after injection, and gradually decreased over the following 3 weeks. Knowledge of the duration for which a specific concentration is maintained is essential to determine when irradiation should be started and how long the tumor should be irradiated. The results of the present study suggest that PDT is most effective on 5 days after the injection of LP-ICG-C18, and that irradiation would be ineffective if provided 3 weeks after the injection. As shown in Fig 4, the intensity of fluorescence was strong at the tumor surface. Moreover, the absence of blood vessels in the tumor was determined by HE staining. We believe that LP-ICG-C18 permeated into the center of the tumor from the surrounding muscular vessels via the EPR effect. If new tumor vessels form within the tumor, we believe that a greater amount of LP-ICG-C18 would accumulate within the tumor. Because LP-ICG-C18 is pegylated, small amounts of LP-ICG-C18 accumulation in the liver, spleen, and other organs were noted in the present study. Although we did not investigate the systemic toxicity of PDT with LP-ICG-C18 injection, this should be evaluated in future studies to ensure the safety of this treatment in humans.

Although we expected that PDT with LP-ICG-C18 would cause cell apoptosis through the generation of singlet oxygen and heat *in vitro*, it is difficult to measure singlet oxygen *in vivo*. However, we observed that PDT with LP-ICG-C18 generated heat in the murine tumors (Fig 6), and we therefore believe that singlet oxygen may have also been generated in these tumors. Some previous studies have measured the levels of singlet oxygen *in vivo*, using either direct or indirect methods [33, 34]. Using such techniques, future studies will be able to measure the extent to which PDT with LP-ICG-C18 generates singlet oxygen generation in tumors.

Although we did observe an antitumor effect of PDT in the present study (Fig 7), the effect was not as substantial as we had expected from the results of the *in vitro* experiments. Since NIR light has been reported to reach a depth of approximately 1 cm [35], we expected that the tumor would be sufficiently irradiated. Apoptotic cells were actually observed in the tumor (Fig 7). The findings shown in Fig 7 may be explained by the weak power from the light source, the short irradiation time, and the low concentration or amount of LP-ICG-C18. By improving these factors, we believe that PDT with LP-ICG-C18 could become more effective.

PDT is limited by the inaccessibility of deep-seated tumors to NIR light. Indeed, NIR light can only reach a depth of 1 cm. Accordingly, it would be difficult to apply PDT to metastatic tumors. However, we expect that it would be possible to apply PDT with LP-ICG-C18 as an endoscopic treatment.

## Conclusions

LP-ICG-C18 is a novel photosensitizing liposome that has been developed at our research group [17]. In this study, PDT with LP-ICG-C18 shows high cytotoxicity, and that LP-ICG-C18 itself is biocompatible under *in vitro* conditions. Furthermore, we observed that LP-ICG-C18 accumulated in the tumor through the EPR effect, and that PDT with LP-ICG-C18 had a good antitumor effect and induced cell apoptosis *in vivo*. Thus, we believe that LP-ICG-C18 could provide effective contributions to the diagnosis and treatment of early esophageal, oral, and pharyngeal cancer (by endoscopy). Furthermore, this therapy could contribute to the treatment of other types of cancer, such as those of the stomach, colon, and bile duct.

In addition, a remarkable characteristic of LP-ICG-C18 is that the ICG-C18 is anchored at the surface of the liposome, which enables various substances, such as medicines, photosensitizers, and RNA, to be loaded within it. Therefore, these substances, which were loaded in LP-ICG-C18, could be released by irradiation on LP-ICG-C18. Thus, LP-ICG-C18 has great potential for clinical applications.
